# Patient characteristics and reasons for discontinuation in a cardiovascular risk management programme in The Netherlands

**DOI:** 10.1186/s12875-024-02293-9

**Published:** 2024-02-11

**Authors:** Geert H.J.M. Smits, Sander van Doorn, Michiel L. Bots, Monika Hollander

**Affiliations:** 1 Julius Center for Health Sciences and Primary Care, University Medical Center Utrecht, Utrecht University, Utrecht, The Netherlands; 2 Primary Care Group PoZoB, Bolwerk 10-14, Veldhoven, 5509 MH The Netherlands

**Keywords:** Primary health care, Cardiovascular risk management, Integrated care, Prevention

## Abstract

**Background:**

Since 2010, an increasing number of patients have participated in a nurse-led integrated cardiovascular risk management programme in the Netherlands. Because it is important to understand which patients discontinue and why, when evaluating the effectiveness of the care programme, the aim was to identify the reasons for discontinuation.

**Methods:**

Electronic health records of 3997 patients enrolled in a nurse-led integrated cardiovascular risk management programme that started on April 1^st,^ 2010, were manually scrutinized for reasons for discontinuation between April 1^st,^ 2010, and April 1^st,^ 2018. In addition to death and moving to a diabetes care programme, we describe 7 different reasons why patients discontinued the programme and compared the patient characteristics of those who discontinued the programme without specific reasons with those who remained in the care programme for 8 years.

**Results:**

Between April 1^st,^ 2010, and April 1^st,^ 2018, 1,190 participants (29.8%) discontinued the CVRM care programme, of whom 271 participants died (6.8%) and 195 were transferred to a diabetes care programme (4.9%). The remaining 724 patients (18.1%) participated 5 years before discontinuation. Of these, 67 (9.3%) had a previous cardiovascular event at the start of the programme. In 355 patients, a specific reason for discontinuation was not found. At baseline, these patients less frequently had a history of CVD than those who continued the programme for 8 years (1.7 vs. 22.6%), were younger (62 vs. 67 years), had less registered cardiovascular comorbidity (atrial fibrillation: 1.1 vs. 7.2%; congestive heart failure 0.3 vs. 1.2%; chronic kidney disease 0.0 vs. 4.5%), were more often smokers (13.0% vs. 4.3%) and took blood pressure- and lipid-lowering drugs twice as often.

**Conclusions:**

In our study we observed that participants who discontinued the nurse-led integrated CVRM care programme between 2010 and 2018 without specific reason or on request were younger, without previous CVD, had less cardiovascular comorbidity and were better adjusted to medication. Exploring the patients’ reasons for discontinuation can contribute to an individualized approach to prevent or reduce discontinuation.

## Background

Since 2010, cardiovascular risk management (CVRM) in the Netherlands has been increasingly organized by primary care groups [1]. In 2020, 1.2 million patients participated in an integrated CVRM care programme organized by 86 primary care groups (https://ineen.nl/wp-content/uploads/2021/06/Benchmark-Transparante-Ketenzorg-2020.pdf). Care groups support general practices with integrated CVRM care executed by a practice nurse (PN) who has her own consultation hours and guides eligible patients 1–4 times a year with lifestyle changes and medication adjustments in close collaboration with the general practitioner (GP). Prior consent of all participants was requested and obtained before the start of the programme. To the best of our knowledge, it is unknown how often and why participants discontinue a long-term CVRM care programme.

Most research on the topic has been performed in patients with a recent cardiovascular event participating in outpatient cardiac rehabilitation programmes (CRPs) in secondary care. Factors associated with discontinuing CRP were smoking, high body mass index (BMI), physical inactivity, cerebrovascular comorbidity, anxiety and depression and the patients’ belief to handle their own problems [2–4], while among the positively associated factors for adherence were age > 65 years, psychological and medication counselling, assessment for patient satisfaction, relaxation therapy and diet classes [5]. Patients withdrawing prematurely from CRP had a twice as high risk for a subsequent cardiovascular event afterwards [6]. Moreover, patients who have a prolonged follow-up in primary care after in-hospital CRP adhere better to long-term treatment targets relating to lifestyle and medication than nonparticipants [7, 8]. Unfortunately, we do not know whether these results are generalizable to long-term primary care programmes, particularly the CVRM care programme, in which many patients do not have cardiovascular disease. To the best of our knowledge, it is unknown how often and why participants discontinue a long-term CVRM care programme. Understanding the reasons why patients discontinue can help providers design a program that better meets the wishes and needs of individual patients with consequently better compliance and ultimately a greater reduction in cardiovascular risk. Furthermore, information on discontinuation is helpful when assessing the effectiveness of such a programme. To gain better insight into the discontinuation of a nurse-led integrated CVRM care programme, the aims of the present study were (1) to determine how often participants discontinued and for what reasons and (2) to compare the patient characteristics of those who discontinued the programme without a specified reason with those who continued the programme for 8 years.

## Methods

### Setting

PoZoB is a primary care group in and around Eindhoven, southeast of the Netherlands, that supports affiliated GPs with the organization of 5 chronic care programmes: diabetes type 2 (DM2), chronic obstructive pulmonary diseases (COPD), cardiovascular risk management (CVRM), psychiatric diseases and fragile elderly. The care group started with the implementation of a nurse-led integrated CVRM care programme on April 1^st,^ 2010, which has been described in detail elsewhere [9]. Practices affiliated with the care group are located in rural, semirural and urban settings comparable with other regions in the Netherlands and therefore can be considered representative. In 2018, the steering committee of PoZoB requested a thorough evaluation of adherence to the CVRM care programme thereby defining our follow-up period to a maximum of 8 years.”

### Study design and study population

**Fig. 1:**
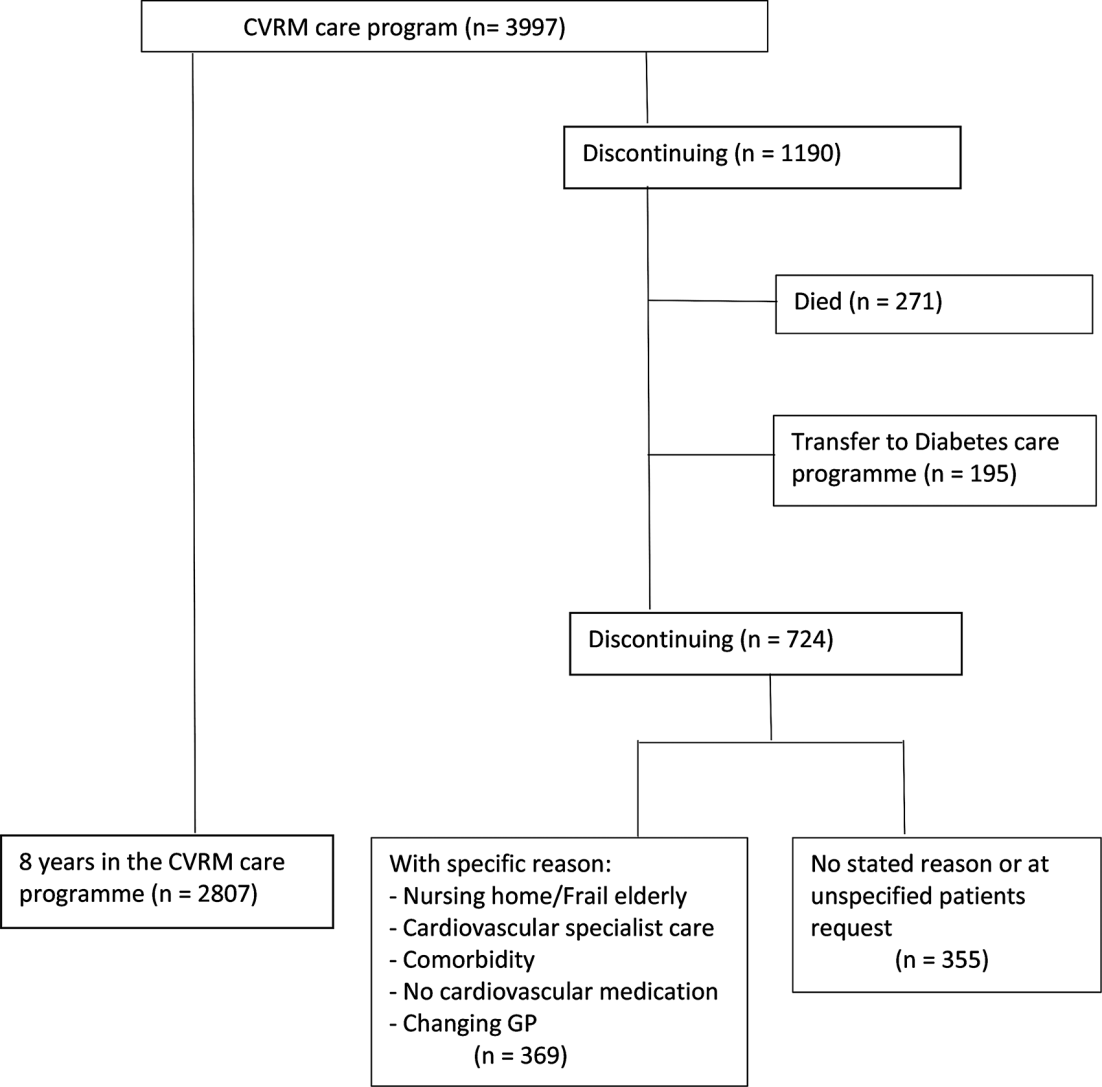
Participants in the study

We performed a study using data from 13 practices (22 GPs, 46,642 registered patients) that had initiated an integrated CVRM care programme on April 1^st,^ 2010, with follow-up until April 1^st,^ 2018. In total, 4260 patients were eligible for the CVRM care programme, of whom 263 declined participation, leaving 3997 patients in this analysis (Fig. 1). Eligibility for the integrated CVRM care programme for both patients at high risk for cardiovascular disease (CVD) as well as for patients with a previous CVD was based on the 2006 CVRM guidelines of the Dutch Society of General Practice [10].

### Data collection

One author (GS) manually scrutinized 3997 electronic health records (EHRs) of patients who enrolled in the nurse-led integrated CVRM care programme on April 1^st,^ 2010. We registered whether patients discontinued the programme before April 1^st,^ 2018, and for what reason. Furthermore, we collected all new diagnoses of acute myocardial infarction (AMI), transient ischemic attack (TIA), stroke, peripheral artery disease (PAD) with invasive or non-invasive treatment and abdominal aortic aneurysm (AAA) with invasive or non-invasive treatment that were recorded with the International Classification of Primary Care (ICPC) codes to distinguish between discontinuation in patients with and without an experienced cardiovascular event between April 1st 2010 and April 1st 2018. To only include patients with established with PAD or AAA, patients with suspected mild PAD or AAA with specialist follow-up but without any treatment were excluded. Routine baseline patient care data were registered by the PN in the multidisciplinary registration system (Care2U). These comprised data from history taking, physical examination, laboratory results, and medication prescriptions based on the Anatomical Therapeutic Chemical (ATC) codes.

### Reasons for discontinuing the CVRM care programme

A priori we registered 9 reasons for discontinuation: 1: death; 2: diagnosed with diabetes mellitus and transition to a diabetes care programme; 3: admission to a nursing home or frail elderly programme; 4: returning to cardiovascular specialist care; 5: returning to specialist care in connection to non-cardiovascular comorbidities; 6: high-risk patients not on cardiovascular medication; 7: changing GPs; 8: without stated reason; 9: at unspecified patient request. “Nursing home”, “returning to cardiovascular specialist care”, “no cardiovascular medication”, “changing GPs” and “comorbidity” were considered discontinuation with a specific reason, while “without stated reason” and “unspecified patient request” were considered discontinuation without a reason. An overview is given in Table 1.

**Table 1:** Reasons for discontinuing the CVRM care program

Reasons for discontinuing (n, %)	Discontinuing in total population (*n* = 3997)*	Discontinuing in patients with previous CVD (*n* = 855)	Discontinuing in patients on high risk for CVD (*n* = 3142)
Died (%)	271 (6.8)	105 (2.6)	166 (4.2)
Diabetes Care Program (%)	195 (4.9)	51 (1.3)	144 (3.6)
Frail elderly program/Nursing home (%)	123 (3.2)	45 (1.2)	78 (2.0)
Cardiovascular specialist care (%)	64 (1.6)	3 (0.4)	61 (1.5)
No cardiovascular medication (%)	81 (2.0)	0 (0.0)	81 (2.0)
Comorbidity (%)	23 (0.6)	1 (0.03)	22 (0.6)
Changing GP (%)	78 (2.1)	12 (0.4)	66 (1.7)
Without stated reason (%)	158 (4.0)	0 (0.0)	158 (4.0)
Unspecified patients request (%)	197 (4.9)	6 (0.02)	191 (4.8)
Total (%)	1190 (29.8)	223 (5.6)	967 (24.2)

*All percentage values in Table 1 refer to the total population (*n* = 3997)

### Ethical considerations

The use of patient data in this study was conducted following privacy legislation in the Netherlands. All patients participating in the integrated CVRM care programme have been informed of the possibility that their anonymized data may be used for evaluation purposes. Data drawn from the patient EHR contained only age and sex, making it impossible for investigators to identify individual patients.

### Analysis

For patient characteristics, categorical variables were reported as numbers and percentages. Continuous variables were reported as the means with standard deviation (SD). Baseline variables of patients who discontinued the programme with and without a specific reason were compared with patients who participated during the full 8 years. No formal statistical testing was performed, as the interest was mainly descriptive and not focused on causality where confounding should be addressed by adjustment. Data were analysed using SPSS (version 22, IBM corp).

## Results

On April 1^st,^ 2010, 3997 patients participated in the care programme, comprising 855 patients (21.4%) with a previous CVD and 3142 patients (78.6%) with a high risk for suffering a CVD in the future. The mean age of the patients was 67.5 years (SD 10.5), and 47% were men. Between April 1^st,^ 2010, and April 1^st,^ 2018, 1190 patients discontinued the CVRM care programme, of whom 271 patients died and 195 patients continued in the diabetes care programme. This left 724 patients discontinuing the CVRM care programme with or without a specific reason, of which 355 patients discontinued with “no reason stated” or “at unspecified request”. These 355 patients participated 4.4 years in the integrated CVRM care programme. The remaining 369 patients discontinued treatment for a number of specific reasons, which are listed in Table 1. Of the 355 patients who discontinued treatment without reason, 349 (98.3%) were classified as having a high risk for CVD, and 6 had a previous CVD (1.7%) (Table 2). When we compared the patient characteristics of those who discontinued treatment without a stated reason and at patient request (*n* = 355) with those who were 8 years in the care programme (*n* = 2807), the following results became apparent. Patients who discontinued treatment were 4.6 years younger at baseline than those who participated for 8 years. Mean systolic blood pressure (SBP), LDL cholesterol and BMI were comparable at baseline: 141.3 mm Hg vs. 141.4 mm Hg, 3.41 mmol/l vs. 3.35 mmol/l and 27.0 kg/m2 vs. 27.9 kg/m2, respectively. Compared to patients who participated for 8 years, those patients who discontinued the programme without reason were more often smokers (13.0% vs. 4.3%) and more often were prescribed blood pressure lowering (BPL) medication (63.7% vs. 25.5%) and lipid modifying (LM) medication (26.2% vs. 11.7%). Furthermore, there were fewer registered comorbidities: atrial fibrillation (AF): 1.1% vs. 7.2%, congestive heart failure (CHF): 0.3% vs. 1.2% and chronic kidney disease (CKD): 0.0% vs. 4.5% in those who discontinued and completed treatment, respectively (Table 2).

**Table 2:** Patient characteristics at baseline (April 1^st,^ 2010)

	8-year CVRM care (*n* = 2807)	Discontinuing CVRM care (*n* = 724)	
		With specific reason (*n* = 369)^a^	Without reason (*n* = 355)^b^
Age (SD)	66.8 (9.7)	71.6 (10.0)	62.2 (11.8)
Years in CVRM care program (SD)	8.0 (0.0)	5.3 (2.0)	4.4 (1.7)
Previous CVD (%)	634 (22.6)	62 (16.9)	6 (1.7)
High risk for CVD (%)	2173 (77.4)	307 (83.1)	349 (98.3)
Men (%)	1311 (46.7)	153 (41.5)	176 (49.6)
Mean LDL-cholesterol (SD)	3,35 (0.9)	3,40 (1.0)	3,41 (1.0)
Mean SBP (SD)	141.5 (17.1)	142.6 (18.4)	141.3 (16.5)
Mean BMI (SD)	27.9 (4.8)	28.3 (6.9)	27.0 (4.7)
Smoking Status (%)			
Yes	4.3	4.2	13.0
No	39.7	35.8	36.0
Before	8.6	6.9	11.8
Not registered	51.1	49.2	39.4
Cardiovascular comorbidity registered (%)^c^			
AF	7.2	5.3	1.1
CHF	1.2	1.1	0.3
CKD	4.5	5.0	0.0
Cardiovascular medication registered (%)^d^			
BPL-medication	25.5	39.7	63.7
LM-medication	11.7	12.7	26.2

^a^With reason: Nursing home, Return to cardiovascular specialist, No cardiovascular medication, Comorbidity, Changing GP

^b^Without reason: No stated reason or unspecified patient request

^c^Comorbidity: AF: atrial fibrillation; CHF: congestive heart failure; CKD: chronic kidney disease

^d^Cardiovascular medication: BPL: blood pressure lowering; LM: lipid modifying

## Discussion

In this study, we evaluated the discontinuation of patients in a nurse-led integrated CVRM care programme and the reasons and patient characteristics associated with discontinuation. Patients who discontinued the care programme without stated reason or at unspecified patient request seemed generally younger, nearly all without a previous cardiovascular disease, had less registered cardiovascular comorbidity, were more often smokers and took twice as much blood pressure lowering and lipid modifying medication at baseline compared to participants who stayed 8 years in the programme.

### Comparison with literature

Although 1.2 million patients participated in a nurse-led integrated CVRM programme in the Netherlands in 2020, to the best of our knowledge, no studies have been published on the frequency and reasons for discontinuation after being enrolled in a long-term CVRM care programme (https://ineen.nl/wp-content/uploads/2021/06/Benchmark-Transparante-Ketenzorg-2020.pdf). Badenbroek et al. evaluated reasons why patients did not participate in a health check and, if they did, why they did not visit their GP despite the estimated increased risk. More than half of their nonresponders thought they did not have a cardiovascular disease, and a quarter had forgotten to make an appointment or reported having no time [11]. A personal approach, more information on cardiovascular diseases, their risks and consequences and the value of health checks were among the most important reasons to reconsider nonparticipation [12]. Much research on nonparticipation and discontinuation comes from studies in participants of longer term outpatient cardiac rehabilitation programmes using a multifactorial approach, targeting unhealthy lifestyle behaviour and medication adherence to reduce the risk of a recurrent CVD [13]. While lack of interest or the notion that the program is not beneficial were often mentioned reasons for nonparticipation [4, 14, 15], discontinuation was often associated with older age [4, 13], female biological sex, [16–18], comorbidity [19], unemployment [18], lower education, a sedentary lifestyle, being divorced or single [20] and smoking [21]. A longitudinal study by Rao and colleagues, however, reported lower adherence to CRP in patients younger than 55 years [22]. Adherence to a cardiac rehabilitation programme, which usually takes a limited number of sessions within 3 months, is, however, difficult to compare with a lifelong programme in primary care with guidance once or twice a year. In our study, patients who discontinued treatment without a stated reason or at unspecified request participated in the programme for an average of 4.4 years. It is possible that patients might have found their own way to implement lifestyle improvements in daily life activities [4, 23]. It could also be very well that the 355 patients who discontinued without stated reason or at request perceived continuation as not beneficial and inconvenient with working hours after a number of years because 230 of them (65%) were 65 years or younger, although being employed is known as a reason for better adherence [14, 18]. A German study on participants in a regional disease management programme in primary care for patients with diabetes observed that those who were younger and employed were more at risk for dropping out [24]. Furthermore, nearly all patients who discontinued treatment without reason or at request were without cardiovascular disease (98.3%). They were, in fact, asymptomatic, which might well be a reason for discontinuing CVRM care because the necessity to continue is perceived as less urgent. This notion is supported by the study of Gucciardi et al., where asymptomatic patients or patients with few symptoms tended to drop out more often than symptomatic diabetic patients [25]. If death and transfer to the diabetes care program are not taken into account, 724 participants (18%) discontinue with the CVRM care program within 8 years, making it an annual discontinue rate of 2,25%. The aforementioned German study reported a 5.5% drop out within less than 3.5 years [24]. A meta-analysis with 41 randomized controlled trials on diabetes management interventions with a follow-up time between 1.5 and 48 months reported a drop-out rate ranging between 1.1% and 39.8% [26]. The results of this meta-analysis, however, comprised data from clinical trials and might not be comparable with data from real-world studies such as ours.

### Potential implications for routine care

Based on the observations in our study, it could be considered to offer less frequent follow-up to younger participants without a previous CVD, those well-adjusted to medication and on target for systolic blood pressure and LDL cholesterol and smoking to increase their motivation to continue with integrated CVRM care. Alternatively, cardiovascular risk factor information could still be collected from those dropping out during regular consultation hours with the GP when the patient, for another non-vascular reason, comes to visit the GP, particularly since in the Netherlands, patients generally visit their GP once every 3–5 years [27]. Alternative data collection may continue with automated calls for blood tests and involving patients in self-management activities such as home blood pressure measurements and weight measurements, thus minimizing direct involvement of the practice. An additional advantage may be that a differentiated approach to participants leads to less work pressure in primary care as a result of the annual increasing number of participants in the CVRM care programme. A potential role for the pharmacy may be explored, since a substantial proportion of those who discontinue used lipid- or blood pressure-lowering medication. Finally, it is important to explore patient motivation for discontinuing the CVRM care programme. If we know the patient’s personal values and beliefs about the necessity of participation, we will be able to motivate them more effectively [28].

### Implications for future research

Loss of follow-up may bias the findings when studying the long-term effect on achieved risk factor level, medication prescription and/or cardiovascular disease incidence. Our findings point towards a potential for bias when studying controlled risk factor levels after 8 years or over. As a considerable percentage of those lost to follow-up had controlled risk factor levels, the remainder of the participants will have a lower percentage of controlled levels, attenuating the potential effect of a programmatic approach.

### Strengths and limitations

To our knowledge, this is the first study that examined discontinuation from a nurse-led integrated cardiovascular risk management programme in primary care in the Netherlands. We were able to carefully scrutinize possible reasons for discontinuation in a large sample of Dutch patients receiving programmatic CVRM care. Second, this patient sample was largely unselected, covered both urban, suburban and rural areas in the Netherlands and therefore in itself was representative of the Dutch population.

It is important to evaluate discontinuation because (i) long-term participation is an indicator for the success of the program and (ii) if we understand why participants discontinue, we may be able to better tailor the programme to the patients’ individual preferences.

A limitation is that we could not further specify the reasons “without stated reason” and “at unspecified request”. This would have given us better insight into the patients’ motivation for discontinuation, but it calls for registration of that information at the source. Unfortunately, no further information was available on the patients’ self-management skills or successful lifestyle improvements before discontinuing the care program, and we did not have information about the patients’ private situation, such as education level or marital status. In addition, we did not have information on depression and anxiety, as it is known that these comorbidities lead to discontinuation and reduced motivation to change lifestyle behaviour [29]. Lastly, the large and representative sample notwithstanding, the descriptive nature of this study warrants caution when interpreting differences in patient characteristics between patients who continue the program and those who discontinue.

## Conclusion

In our study we observed that participants who discontinued the nurse-led integrated CVRM care programme between 2010 and 2018 without specific reason or on request were younger, without previous CVD, had less cardiovascular comorbidity and were better adjusted to medication. Exploring the patients’ reasons for discontinuation can contribute to an individualized approach to prevent or reduce discontinuation.

## Data Availability

Anonymized patient data derived from the multidisciplinary information system (Care2U) can be send by mail on request: g.smits@pozob.nl.

## Abbreviations

CVRM: Cardiovascular risk management
DM2: Diabetes type 2
COPD: Chronic obstructive pulmonary disease
PN: Practice nurse
GP: General practitioner
CRP: Cardiac rehabilitation programme
BMI: Body mass index
PoZoB: Praktijkondersteuning Zuidoost Brabant
CVD: Cardiovascular disease
AMI: Acute myocardial infarction
TIA: Transient ischaemic attack
PAD: Peripheral artery disease
AAA: Aortic abdominal aneurysm
EHR: Electronic health record
ATC: Anatomical therapeutic chemical
SD: Standard deviation
BPL: Blood pressure lowering
LM: Lipid modification
AF: Atrial fibrillation
CHF: Congestive heart failure
CKD: Chronic kidney disease
